# Retrograde intrarenal surgery for lower pole renal calculi smaller than one centimeter

**DOI:** 10.4103/0970-1591.44265

**Published:** 2008

**Authors:** Hemendra Navinchandra Shah

**Affiliations:** Department of Urology, R. G. Stone Urology and Laparoscopy Hospital, 21-A, 14-A Road, Ahimsa Marg, Khar (W), Mumbai - 400 052, India

**Keywords:** Flexible ureteroscopy, holmium laser lithotripsy, lower calyx, management, renal calculi, retrograde intrarenal surgery

## Abstract

**Objectives:**

Recently there has been an increasing interest in the application of retrograde intrarenal surgery (RIRS) for managing renal calculi. In this review we discuss its application for the management of lower calyceal (LC) stones less than 10 mm in maximum dimension.

**Materials and Methods:**

Literature was reviewed to summarize the technical development in flexible ureterorenoscopy and its accessories. Further, the indications, outcome and limitations of RIRS for LC calculi < 1 cm were reviewed.

**Results:**

Use of access sheath and displacement of LC stone to a more favorable location is increasingly employed during RIRS. Patients who are anticoagulated or obese; those with adverse stone composition and those with concomitant ureteral calculi are ideally suited for RIRS. It is used as a salvage therapy for shock wave lithotripsy (SWL) refractory calculi but with a lower success rate (46-62%). It is also increasingly being used as a primary modality for treating LC calculi, with a stone-free rate ranging from 50-90.9%. However, the criteria for defining stone-free status are not uniform in the literature. The impact of intrarenal anatomy on stone-free rates after RIRS is unclear; however, unfavorable lower calyceal anatomy may hamper the efficacy of the procedure. The durability of flexible ureteroscopes remains an important issue.

**Conclusions:**

RIRS continues to undergo significant advancements and is emerging as a first-line procedure for challenging stone cases. The treatment of choice for LC calculi < 1 cm depends on patient's preference and the individual surgeon's preference and level of expertise.

## INTRODUCTION

Treatment of lower pole calyceal (LC) stones presents a dilemma for the urologist.[1] Extracorporeal shock wave lithotripsy (SWL) is a technology that relies on spontaneous passage of the fragment to achieve a stone-free state. Hence, its results have been less than optimal for LC stones and in particular for patients with unfavorable intrarenal anatomy since these fragments are less likely to clear with SWL.[2] The role of flexible ureteroscopy in the urologist's armamentarium has undergone a dramatic evolution.[3] This is generally attributed to improvements in fiber optics designs, downsizing of instrumentations, better irrigation system and the availability of small instruments, both powered and mechanical to allow complex maneuvers within the confines of the upper urinary tract. Parallel to these developments, there is an increasing interest in application of retrograde intrarenal surgery (RIRS) for treatment of renal calculi. In this review we discuss the technical development in intrarenal surgery and its application for the management of LC stones less than 10 mm in maximum dimension.

## EVOLUTION OF FLEXIBLE URETERO-RENOSCOPES AND ITS IMPLICATION IN TREATMENT OF LOWER POLE CALCULI

Intrinsic limitations of the deflection capabilities of the single-deflection ureteroscope limit their ability to execute the difficult angles necessary to gain access to many LC stones. In addition, even when the ureteroscope can be maneuvered into the LC and the stone is located, the placement of instruments or laser fibers in the working channel can decrease the maximal angle of deflection and prevent further access or examination of the stone burden.[4] Landman *et al*. reported a failure rate of 21% and 42% due to inability to access the LC effectively.[5] This limitation of ureteroscopy in the management of LC stone disease has led to the development of a dual deflection ureteroscope. With a second, more proximal, unidirectional deflection point controlled with a separate lever, this ureteroscope has the ability to achieve greater overall deflection and thus may be of significant benefit in the management of LC stone disease. Another advantage of the dual deflection ureteroscope is that they allow use of larger instruments in the working port with a smaller impact on overall deflection. Shvarts *et al*., found that nitinol baskets, 200 µ and 360µ laser fiber decrease the maximal deflection angle by 4.4, 9.9 and 27.7% respectively.[4] It is important to remember that 500 µ laser fiber is not recommended to be used with flexible ureteroscope due to risk of fiber breakage and ureteroscope damage.

Ames *et al*., studied the impact of various available nitinol baskets on ureteroscope channel flow and deflection and found that average baseline irrigant flow (46.6 ml/min) decreases by 78.5% to 9.9ml/min with the smaller baskets (Microvasive 1.9F and Cook 2.2 F) and by 99.1% to 0.4 ml/min with larger baskets (ACMI 3F and Microvasive 3.0F).[6] This decrease in irrigant flow causes deterioration in visibility especially if debris or bleeding is present.[6] For this purpose unsheathed nitinol baskets (naked basket concept) were employed which allowed an additional 15− 20° of active deflection and a 2-30 fold increase in irrigant flow.[7]

Recently, two new-generation flexible ureteroscopes, the Flex-X (Karl Storz) and the DUR-8 Elite (ACMI) have been introduced with a crush-resistant flexible shaft, and dual 270° deflection.[8] DUR-8 Elite has a second active deflection located more proximally on the shaft, allowing a maximum deflection of > 270° as well as an S-shaped deflection. However, such high deflections enhance friction within the working channel which can resist opening of a stone basket with maximum deflected tip. In addition, maximum deflection increases the risk of iatrogenic trauma in the narrow collecting system. Consecutive bleeding or perforation may impair treatment outcome.

Digital flexible ureteroscope is the latest evolution in RIRS. These ureteroscopes (DUR-D, Gyrus ACMI) integrate the endoscope, digital camera and the light source. This obviates the need for a separate camera head since the scope has a digital camera chip (CCD or CMOS) mounted on the tip of the ureteroscope. Since these devices do not require a separate light cord or camera head, there is a potentially prolonged lifespan. The DUR-D image has no pixilation, glare or moiré effect.

## PROCEDURE

Anesthesia- General anesthesia is preferred so that the movement of kidney with respiration can be controlled.

Position- Patient is usually placed in a modified combined Trendelenburg (head down approximately 20°) lithotomy position.[9] Prone head-down position (20°) facilitates access to the LC infundibulum and its minor calices, especially in obese patients.[10]

### Methods of introducing flexible ureteroscope

1. Traditional (Railroading) method- In this method, a double lumen ureteral catheter is used to introduce two guide wires in the pelvicaliceal system. The ureteroscope is then backloaded over a second guide wire and advanced up the ureter under fluoroscopic guidance. Difficulty during passage may be encountered at the ureteral orifice, the ureterovesical junction, or anywhere along the middle and proximal ureter secondary to ureteral spasm. Guidewire trauma to the working channel may shorten the lifespan of these fragile instruments. Hence, the flexible ureteroscope should not be backloaded onto an Amplatz superstiff guide wire.[3] Special guide wire is available with floppy tip on both ends. This can be safely used to backload ureteroscope.
2. Passage through the cystoscope sheath- This is a modification of railroad technique in which a flexible ureteroscope is introduced over a working guide wire in railroad fashion through the lumen of the cystoscope sheath. This avoids buckling of the ureteroscope at the ureterovesical junction. However, there is a possibility of damaging the fragile sheath of the ureteroscope on the tip of the rigid cystoscope sheath.[3]
3. Ureteral access sheath- Ureteral access sheath provides an effective and reliable ureteral access for flexible ureteroscopy. It is ideal for situations where multiple passages of the ureteroscope are anticipated since it allows rapid entry and re-entry into the collecting system.[3] Newer-generation access sheaths have an impregnated wire to make them kink-resistant, and hydrophilic coating making them safer and easier to insert. The kink-resistant sheath also prevents the problem of bladder buckling and decreases the wear and tear of the ureteroscope.[3] Recently, access sheaths are manufactured with a dual lumen system for the use of irrigation, contrast instillation or instrument insertion. Whether these will translate into any added clinical benefit is yet to be confirmed.
  The use of access sheath is associated with a few potential advantages in RIRS. The efflux of irrigant fluid through the access sheath around the ureteroscope optimizes visibility while maintaining low intrapelvic pressure. Hence, the irrigant can be pressurized to 100 to 200 mm Hg, which greatly enhances vision without raising intrapelvic pressure above 40 cm H_2_O. In addition this rapid flow of irrigant helps to flush smaller stone particles out of the collecting system, allowing them to exit the sheath.[3] Auge BK used hand irrigation during ureteroscopy in five patients who had percutaneous nephrostomy (PCN) tube *in situ*.[2] They found that the mean pressure within the collecting system, with the ureteroscope in the renal pelvis without the use of access sheath was 94.4 mm Hg and the same reduces to 40.6 mm Hg with the use of access sheath.[11] Hence the access sheath is potentially protective against pyelovenous and pyelolymphatic backflow. This may have clinical implication during ureteroscopic treatment of struvite calculi or calculi associated with urinary tract infection (UTI) and also upper tract tumors. L'esperance *et al*., found that the use of ureteral access sheath improved stone-free rates.[12] Overall stone-free rates with and without the use of access sheath were 79% and 67% respectively.
  However, in porcine model, the ureteral blood flow declined significantly during ureteral access sheath use. Hence, opponents argue that the use of access sheath which carries a risk of long-term ureteral stricture formation.[13] However, there are no reported clinical cases of ureteric stricture development attributable to use of access sheath. Another concern about access sheath use is its additional cost. However, Kourambas *et al*. found that its use decreased operative time by 10 min and also decreased requirement of balloon dilatation of the ureteric orifice. This counterbalanced the additional expense of the access sheath.[13]
4. Wireless ureteroscopy-It involves passage of flexible ureteroscope into the ureter like a ureteric catheter without the use of a guide wire. In a large study, 227 patients were successfully ureteroscoped using this technique.[14]
5. Passive ureteral dilatation with pre-RIRS DJ stenting- Although it is a very useful maneuver for safe introduction of flexible ureteroscope, it is associated with morbidity of an additional procedure and that of a ureteric stent.[14] It is particularly useful in patients with tight ureter precluding active ureteral dilatation and ureteroscopy.

### Mapping of collecting system and access to calyces

**Figure 1:**
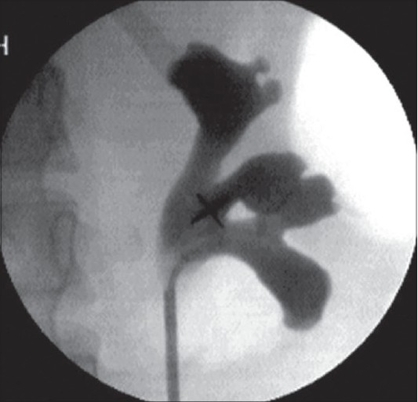
Retrograde pyelogram provides a road map for the flexible ureteroscope to enter desired calyx

Once the ureteroscope is advanced through the pelviureteral junction, the renal pelvis is inspected and infundibula located. The visual image is coordinated with a fluoroscopy image to enter appropriate calyces [Figure 1]. Care should be taken to avoid over-advancement of the ureteroscope under deflection since this can damage endoscope fibers or deflection mechanism. Manipulations of ureteroscope within the collecting system consist of six movements: advancement, withdrawal, rotation in either direction, indeflection or undeflection.[15]

### Methods of stone retrieval

**Figure 2:**
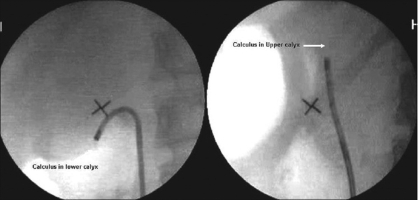
Technique of calculus displacement: Nitinol tipless basket is used to relocate a calculus from lower calyx into a more favorable upper calyceal location for holmium laser lithotripsy

Smaller stones can be grasped in a basket or stone-graspers and removed intact. Three- pronged stone-grasping forceps are the safest instruments for removing calculi.[16] They permit disengagement of calculi that are found to be too large to be safely removed from the ureter. This is important in RIRS, since there is no second channel to permit fragmentation of an unyielding stone trapped within a basket. Larger stones need fragmentation using intracorporeal Holmium laser lithotrity or electrohydraulic lithotripsy. Holmium laser is absorbed for 3 mm in water and 0.4 mm in tissue, causing fragmentation by photo thermal reaction with the crystalline stone matrix. It has become the intracorporeal lithotripsy device of choice. However, *in situ* fragmentation of stone is not possible in 28-34% of LC stones because of the reduction in deflection of the ureteroscope with the laser fiber in place, thereby precluding reentry into the LC.[2] To counteract this difficulty, the technique of calculus displacement using nitinol baskets and graspers from LC into upper pole calyx was described.[2,12] The calculus displacement to a more favorable position was associated with better stone-free rates [Figure 2]. Schuster TG confirmed these findings. It is reported that a 200-µ laser fiber decreases ureteroscope deflection by 7-16%. Deflecting the calculus into a more accessible calyx eliminates this problem, allows easier manipulation of the ureteroscope and decreases the likelihood of unintentionally leaving residual stone fragments that have fallen out of camera view and are inaccessible with retrograde flexion. In addition small residual fragments left after successful intracorporeal lithotripsy may pass more easily out of the kidney from the upper or mid-calyceal system than from a lower pole.[17] In view of the same, usually all LC calculi are relocated using tipless nitinol basket or gravitational drift.[9,18]

## ROLE IN CLINICAL PRACTICE

Patients who are fully anticoagulated, obese or SWL failure; those with adverse stone composition (calcium oxalate monohydrate or Cystine); and those with concomitant ureteral calculi may be ideal candidates for an attempt with ureteroscopic treatment of the LC stones.[2] A recently published series confirmed that ureterorenoscopy and holmium YAG lithotripsy can be performed safely and efficaciously for renal calculi in patients on anti-coagulation therapy.[18] It has no ill effects on renal function in the patients with mild to moderate renal insufficiency.[19] In eight morbidly obese patients treated with RIRS, 70% stone-free rate was observed after a single treatment.[10] There were no procedure-related complications. Patients undergoing ureteroscopy for ureteral calculi who have concurrent, ipsilateral, small LC stones may best be served by the simultaneous treatment of the renal calculi ureteroscopically rather than asynchronous treatment with SWL. In a study by Hollenbeck *et al*., 91% patients were stone-free after treatment of both ureteral and ipsilateral LC calculi.[20]

RIRS is a preferred method of stone treatment in pilots. They have to be completely stone-free before resuming their job to prevent sudden in-flight incapacitation. In a retrospective study of aviation pilots with urinary calculi Zheng *et al*., found that stone-free rate of endoscopic procedure was 100% as compared with 35% for those treated with SWL.[21] They showed that the average number of work weeks lost for SWL, percutaneous nephrolithotomy (PCNL), and ureteroscopy were 4.7, 2.6, and 1.6 respectively.

RIRS is often used as a salvage therapy after SWL failure, assuming that the ureteroscopic technique will be mostly indifferent to the factors that lead to poor stone fragmentation or a low likelihood of spontaneous passage. However, this assumption is not well supported in the literature [Table 1].[22,25] The low success rate of RIRS (46-62%) in SWL refractory renal calculi was attributable to the anatomic features that contribute to SWL failure.[22] In patients treated with more than one session of SWL, a partially fragmented stone may become embedded in the renal mucosa and can also result in SWL failure.

**Table 1:** Summary of published series of retrograde intrarenal surgery for refractory renal calculi

Authors (Year published)	Total N. of patients (LC cal.)	Stone size (mm)	OR time	Postoperative imaging	Stone-free rate	Complications	Comments
Menezes P (1999)[22]	37 (14% LC)	< 10 mm in 40 patients	45	abdominal plain film	62% at 3 months Small asymptomatic fragments- 13%	1- pyelonephritis	Treatment failure- 25%
Stav K (2003)[23]	81 (31 LC)	9.2	110	Sonography and abdominal plain film	46% stone-free 67%-including < 3 mm fragments	9- failure to reach stone with laser fiber *in situ* 5- extravasation or bleeding 6-UTI	Lower pole stones and larger stone size are difficult to treat
Jung HJ (2006)[24]	38 (16 LC)	9	-	IVP	58% 11%- < 4 mm fragments	No major	Lower pole stones and larger stone size are difficult to treat
Holland R (2006)[25]	51 (60% LC)	8.7	103	Sonography and abdominal plain film	51% 67%- CIRF At 11/2 to 3 months	1- ureteral perforation 2- UTI	Lower stone-free rate wrt primary RIRS (67% vs. 80% including CIRF)

OR- operation room; UTI- urinary tract infection; CIRF- clinically insignificant residual fragments

RIRS is increasingly used as a primary modality for treating LC calculi [Table 2].[1,8,9,17,20,26–29] It is associated with a stone-free rate ranging from 50-90.9%. However, the criteria for defining stone-free status was not uniform amongst various published series in the literature [Table 2]. In a multicentric prospective randomized trial, Pearle MS *et al*., failed to demonstrate a statistically significant difference in stone-free rates between SWL and ureteroscopy for the treatment of small LC calculi. Although ureteroscopy was associated with higher stone-free rates (50% vs. 35% for SWL) and fewer procedures per patient, the patient preference were higher for SWL.[28] In a postal and internet survey of American urologists in 2003, 88% preferred SWL for < 1 cm LC calculi. The reasons may include the ease of performing SWL; lack of availability of flexible ureteroscope, lack of training in advanced endourological techniques or the belief that small residual fragments after SWL may not be of clinical significance.[30]

**Table 2:** Published series of retrograde intrarenal surgery for < 1 cm lower calyceal calculi

Authors (year published)	patients with LC cal.	Stone size (mm)	OR time.	Postoperative imaging	Stone-free rate	Complications	Comments
Grasso M (1999)[26]	47	< 10	38	Sonography and abdominal plain film	82% 7%- < 4 mm 5% ≥ 5 mm	8% - needed stenting for renal colic 4%- Symptomatic UTI	First article to address issue of RIRS for LC calculi. Long (> 3 cm) infundibulum and Infundibular stenosis associated with high failure rates. Failure of endoscopic access 6%
Tawfiek ER (1999)[27]	23	7 (3- 18)	90*	abdominal plain film	87% at 3 months (include < 3 mm fragments)	Rare and include fever and stent-related symptoms.	Good for intermediate size stones 95% patients were treated on outpatient basis.
Hollenbeck BK (2001)[20]	52	7.1	64	abdominal plain film	89% at 2.7 months includes second stage procedure	8%. Include readmission for pain.	Stent needed in 61% patients Second procedure needed 6% patients
Schuster TG (2002)[17]	95	8 and 10.3	64 and 80	abdominal plain film	61 and 79% overall For stone < 1 cm 77 and 89%.	1- minor post-operative complications. 1- Bleeding obstructing visual field.	First study to evaluate impact of displacement technique on stone-free rates.
							Displacement improves stone-free rates in LC calculus 1-2 cm.
Pearle MS (2005)[28]	35	6.9	90.4	CT scan	50% at 3 months 72 % < 4 mm	2 small perforation needing stenting	Multi-institutional RCT comparing outcome of URS with SWL for lower pole calculi < 1 cm. URS less tolerated by patients. Only 63% patients would choose to undergo same procedure again. Failure of endoscopic access 14%.
Portis AJ (2006)[9]	19	9.1	39.3	CT scan	52.9% at 1 month 88.2% (2-mm fragments) 100% (4-mm fragments)	3 out of 58 patients developed ureteric perforation	Stone location did not alter stone-free rates Active stone extraction performed
Wendt-Nordahl G (2007)[8]	32	8	44	Sonography and abdominal plain film	87.5% with new and 81.5% with old ureteroscope (includes up to 3-mm fragments) 100% at 1 month	6.3%-colic 25% hematuria 6.3%- UTI occurred in patients with new ureteroscope.	Compared results of new (270°) ureteroscope with the standard ureteroscope. 24 patients had past SWL.
Perlmutter AE (2007)[1]	44	6.89	-	Predominantly abdominal plain films	90.9% at 3 months	2 out of 84 patients developed ureteric stricture	Stone location did not alter stone-free rate.
Cannon GM (2007)[29]	21	12	-	variable	76% overall and 93% for < 15 mm calculus.	nil	First study to assess outcome of RIRS in pediatric population (mean age 15 years) with LC stones. Additional intervention needed in 8 patients.

* Include all patients with RIRS; LC- lower calyceal; URS- ureteroscopy; UTI- urinary tract infection; RIRS- retrograde intrarenal surgery; RCT- randomized control trial; CT-computerized tomography

## IMPACT OF INTRARENAL ANATOMY ON STONE-FREE RATES

The impact of intrarenal anatomy on the success rate of RIRS is controversial. Long lower pole infundibulum (>3 cm) and infundibular stenosis were statistically significant negative parameters influencing the success of RIRS for lower pole calculi.[26] Elbahnasy *et al*., found 62% success rates in 13 patients treated. They suggested that intrarenal anatomical variants which inhibited SWL had a smaller role in the overall success rate of RIRS.[31] However, contrary to this, RVS Kumar found that acute infundibulo-pelvic angle < 250 was a statistically significant predictor of failure to access LC.[32] A lower pole calyx with acute angle going medially would be extremely difficult or any calyx with narrow infundibulum and acute angle would be difficult to negotiate.

## DURABILITY OF SCOPES

Unfortunately, the flexibility and smaller diameter of the flexible ureteroscope comes with a cost— “endoscopic fragility”. The number of urologists using a single ureteroscope, experience of the endoscopist, location of the pathology, use of accessory instruments, duration of procedure and scope handling in between cases may all play a role in flexible ureteroscope trauma.[33] Historically, the number of procedures performed before a flexible ureteroscope requires repair averaged 6–15. However, by incorporating new ureteroscopic accessories, such as nitinol devices, a ureteral access sheath and the 200 µ holmium laser fiber into common practice, one can reduce the strain on fragile 7.5-F endoscopes, thereby maximizing their longevity.[34] Pietrow *et al*., found that a ureteroscope averaged 27.5 separate operative procedures before being sent for repair. Channel perforation/ moisture in the optics was the commonest cause of ureteroscope breakage followed by poor deflection and scratching of the lens. Channel perforation was directly attributable to damage by a laser fiber in all instances.[33] Hence care must always be exercised when advancing any laser fiber through the working channel, because these fibers are capable of penetrating the wall of instruments if passed while the scope is deflected. Therefore, straightening the tip of the ureteroscope will allow for easy passage of the laser fiber before manipulating the ureteroscope into the LC.[34]

## CONCLUSIONS

RIRS is a relatively new procedure that continues to undergo significant advancements. It offers the low morbidity of SWL but the potential for stone-free rates approaching those of percutaneous surgery for small to moderate-sized renal calculi. Hence, it is emerging as a first-line procedure for increasing challenging stone cases. The LC of the kidney is the most difficult part of the kidney to access, although with new flexible ureteroscopes the LC can be accessed in 93% of cases. Selection of treatment modality for a LC calculus requires an informed conversation with the patient about the risks and benefits of various procedures and their associated stone-free rates. Patient may choose surgical treatment (RIRS) in order to achieve stone-free status immediately. On the contrary, a patient may choose to treat his or her stone with SWL, accepting a protracted time to achieve stone-free status, in order to avoid the need for a general anesthesia, instrumentation and possibility of a stent after the procedure. The treatment of choice also ultimately depends on the individual surgeon's preference and level of expertise. The literature review suggests that a flexible ureteroscope and holmium laser should be an essential part of the armamentarium at any complete stone treatment centre.
